# Protective effect of ursodeoxycholic acid on COVID-19 in patients with chronic liver disease

**DOI:** 10.3389/fcimb.2023.1178590

**Published:** 2023-05-03

**Authors:** Yanyan Li, Na Zhu, Xinyu Cui, Yingying Lin, Xin Li

**Affiliations:** ^1^Center of Integrative Medicine, Beijing Ditan Hospital, Capital Medical University, Beijing, China; ^2^Center of Integrative Medicine, Peking University Ditan Teaching Hospital, Beijing, China

**Keywords:** coronavirus disease 2019, severe acute respiratory syndrome coronavirus-2, ursodeoxycholic acid, chronic liver disease, infection, propensity score matching analysis

## Abstract

**Objective:**

Ursodeoxycholic acid (UDCA) may reduce susceptibility to severe acute respiratory syndrome coronavirus-2 (SARS-CoV-2) infection by downregulating angiotensin-converting enzyme 2 (ACE2), based on recent experimental investigation. This study aimed to determine the potential protective effect of UDCA against SARS-CoV-2 infection in patients with chronic liver disease.

**Methods:**

Patients with chronic liver disease receiving UDCA (taking UDCA ≥1 month) at Beijing Ditan Hospital between January 2022 and December 2022 were consecutively enrolled. These patients were matched in a 1:1 ratio to those with liver disease not receiving UDCA during the same period by using a propensity score matching analysis with nearest neighbor matching algorithm. We conducted a phone survey of coronavirus disease 2019 (COVID-19) infection during the early phase of the pandemic liberation (from 15 December 2022 to 15 January 2023). The risk of COVID-19 was compared in two matched cohorts of 225 UDCA users and 225 non-UDCA users based on patient self-report.

**Results:**

In the adjusted analysis, the control group was superior to the UDCA group in COVID-19 vaccination rates and liver function indicators, including γ-glutamyl transpeptidase and alkaline phosphatase (p < 0.05). UDCA was associated with a lower incidence of SARS-CoV-2 infection (UDCA 85.3% *vs.* control 94.2%, p = 0.002), more mild cases (80.0% *vs.* 72.0%, p = 0.047), and shorter median time from infection to recovery (5 *vs.* 7 days, p < 0.001). Logistic regression analysis showed that UDCA was a significant protective factor against COVID-19 infection (OR: 0.32, 95%CI: 0.16–0.64, p = 0.001). Furthermore, diabetes mellitus (OR: 2.48, 95%CI: 1.11–5.54, p = 0.027) and moderate/severe infection (OR: 8.94, 95%CI: 1.07–74.61, p = 0.043) were more likely to prolong the time from infection to recovery.

**Conclusion:**

UDCA therapy may be beneficial in reducing COVID-19 infection risk, alleviating symptoms, and shortening the recovery time in patients with chronic liver disease. However, it should be emphasized that the conclusions were based on patient self-report rather than classical COVID-19 detection by experimental investigations. Further large clinical and experimental studies are needed to validate these findings.

## Introduction

Since the liberation of epidemic control on 7 December 2022, there has been a sudden increase in the number of coronavirus disease 2019 (COVID-19) infections in China. Currently, COVID-19 infections were mostly mild due to the attenuated pathogenicity of the virus (Kim et al., 2022). However, the rapid mutation of severe acute respiratory syndrome coronavirus-2 (SARS-CoV-2) and the inability of antibodies to provide broad-spectrum protection among various strains might lead to persistent transmission of COVID-19 (Cao et al., 2022; Suzuki et al., 2022). Therefore, a management strategy based on symptomatic treatment would be a feasible regimen to counter the threat posed by this insidious virus in the post-COVID-19 era.

Ursodeoxycholic acid (UDCA) is used as a cholagogic drug, which may provide health benefits to many illnesses. UDCA has been confirmed to possess protective effects on hepatocytes, including reducing cholestasis, improving liver function, and alleviating hepatic fibrosis (Lindor et al., 2019; Ye et al., 2020). A recent experimental investigation has reported that UDCA could decrease susceptibility to SARS-CoV-2 infection by downregulating angiotensin-converting enzyme 2 (ACE2) (Brevini et al., 2022).

Multiple pieces of evidence revealed that preexisting chronic liver disease was associated with poor prognosis in patients with COVID-19 (Ji et al., 2020; Marjot et al., 2022). Data from Yang et al. suggested that patients with SARS-CoV-2 and chronic hepatitis B co-infection had worse outcomes than those without hepatitis B, including increased intensive care unit (ICU) admission and death (Yang et al., 2022). Moreover, autoimmune liver disease, including autoimmune hepatitis and primary biliary cirrhosis, potentially increased the risk of severe SARS-CoV-2 infection, hospitalization, and mortality due to the patients’ immunocompromised status (Ampuero et al., 2021; Efe et al., 2021). Whether UDCA could reduce SARS-CoV-2 infection risk and disease severity in patients with chronic liver disease was unclear. Herein, we took advantage of the extensive application of UDCA in liver diseases, such as hepatitis B and autoimmune liver disease, to investigate its effect on COVID-19 during the epidemic liberation period.

## Materials and methods

### Patient population

A single-center cohort study was conducted at Beijing Ditan Hospital. Patients with chronic liver disease (hepatitis B associated with cholestasis or liver dysfunction, and primary biliary cirrhosis) receiving UDCA (taking UDCA ≥1 month) from January 2022 to December 2022 were included in the UDCA group. Patients (hepatitis B and autoimmune hepatitis) not receiving UDCA during the same period were enrolled in the control group. Exclusion criteria included the following: 1) repeated COVID-19 infection; 2) complicated with multiple chronic liver diseases; 3) concomitant with other coexisting chronic viral infections, such as cytomegalovirus and acquired immunodeficiency syndrome; 4) accompanied by other severe infections; 5) with active or suspected malignancy or history of malignancy; 6) pregnancy and breastfeeding; 7) age ≤ 18 years.

### Ethical considerations

This study was approved by the institutional review board of Beijing Ditan Hospital (approval number: DTEC-KT2023-001-01) and performed in accordance with the ethical standards laid down in the 1964 Declaration of Helsinki and its later amendments. As this study was conducted during the epidemic, the patients were unable to come to the hospital to sign the informed consent forms, but verbal informed consent was obtained from all patients during the telephone survey.

### Data collection

Demographic variables included age, sex, body mass index, personal history, clinical complications, COVID-19 vaccination, and UDCA administration information. The most recent laboratory investigations prior to SARS-CoV-2 infection were collected for liver function indicators (such as γ-glutamyl transpeptidase, alkaline phosphatase, and albumin), serum lipid parameters triglyceride and total cholesterol levels, blood routine indices (white blood cell, hemoglobin, and platelet counts), glycosylated hemoglobin, and estimated glomerular filtration rate. These variables were collected from electronic medical records.

We then conducted a phone survey to investigate SARS-CoV-2 infection in these patients during the early phase of epidemic liberation (from 15 December 2022 to 15 January 2023). The main data collected were COVID-19 vaccination, SARS-CoV-2 infection, symptom characteristics, treatment, prognosis, time from infection to recovery, and progression of liver disease during COVID-19 infection.

### Definitions and endpoints

Chronic liver disease was defined as the progressive deterioration of liver function for more than 6 months, including the synthesis of clotting factors, detoxification of metabolic waste products, proteins, and the secretion of bile. All patients in this study had a disease duration of more than 6 months.

Overweight/obesity referred to a body mass index greater than 24.

Drinking was defined as current alcohol use or abstinence from alcohol for less than 6 months.

Recovery time referred to the time from infection to symptom disappearance. Day 1 referred to the time when patients reported COVID-19-related symptoms, such as fever and cough.

The diagnosis and classification criteria of COVID-19 referred to the 10th edition of the Chinese protocol for the treatment of SARS-CoV-2 infection (http://www.nhc.gov.cn/). Based on the disease severity, COVID-19 was categorized into four types: mild, moderate, severe, and critical cases.

The primary endpoint was the SARS-CoV-2 infection rate since the liberation of epidemic prevention. Secondary outcome measures were the COVID-19 severity and time from infection to recovery.

### Statistical analysis

#### Propensity score matching analysis

Given the inherent selection bias of patients with different chronic liver diseases, we controlled the confounding by performing a propensity score matching analysis. The propensity score was calculated with an *a priori* logistic regression model based on covariates such as age, sex, body mass index, and clinical complications. Patients with chronic liver disease receiving UDCA were then matched in a 1:1 ratio to those not receiving UDCA. After propensity score matching analysis, we trimmed 204 observations (104 in the UDCA group and 100 in the control group) from the lower and upper tails of the propensity score due to a lack of common support. Propensity score matching was performed using an SPSS-R plugin for R packages (MatchIt, Ritools, and cem).

#### Comparison of variables and logistic regression model analysis

Continuous variables were expressed as mean ± standard deviation (M ± SD) or median (interquartile range) in case of skewed distribution. The difference between groups was analyzed by Student’s *t*-test or the Mann–Whitney U test. Categorical variables were presented as percentages (%), and their statistical analysis was performed by the chi-square test or Fisher’s exact test. Logistic regression analysis was used to determine the factors influencing SARS-CoV-2 infection in the matched cohorts (450 cases) and potential risk factors for recovery time in patients with symptomatic infection in the UDCA group (190 cases). The reference variables were as follows: age < 50 years, no diabetes mellitus, unvaccinated/partially vaccinated, UDCA dose ≤0.75 g/day, UDCA duration <1 year, and mild infection. The results were presented as odds ratio (OR) and 95% confidence interval (CI). Statistical analysis was performed with SPSS (version 26), and figures were generated using GraphPad Prism (version 9.4.1). A two-sided p of less than 0.05 was considered significant.

## Results

### Baseline characteristics

A total of 225 patients with the chronic liver disease treated with UDCA were matched to 225 patients with chronic liver disease not receiving UDCA. Baseline characteristics before and after propensity score matching of the population are listed in **Table 1**. In the matched arms, the mean age was 53 years, and the majority of patients were female. The mean body mass index was 24 kg/m^2^. The proportions of smoking and drinking were relatively low. Approximately one-fifth to one-quarter of the people had at least one chronic complication, such as hypertension and cardiovascular disease. Furthermore, approximately 23% of the patients had varying degrees of cirrhosis.

**Table 1:** Baseline characteristics.

Characteristics	Unadjusted			After propensity score matching		
	UDCA (n = 329)	Control (n = 325)	p-Value	UDCA (n = 225)	Control (n = 225)	p-Value
Personal history						
Age (years)	56.9 ± 12.3	50.9 ± 11.4	<0.001	53.1 ± 12.0	53.6 ± 10.7	0.647
Female, n (%)	234 (71.1)	149 (45.8)	<0.001	134 (59.6)	128 (56.9)	0.566
Body mass index (kg/m^2^)	23.3 ± 3.3	24.3 ± 3.2	<0.001	23.9 ± 3.1	23.9 ± 3.4	0.910
Overweight/obesity, n (%)	110 (33.4)	149 (45.8)	0.001	92 (40.9)	91 (40.4)	0.924
Smoking, n (%)	57 (17.3)	116 (35.7)	<0.001	55 (24.4)	57 (25.3)	0.827
Drinking, n (%)	45 (13.7)	109 (33.5)	<0.001	45 (20.0)	50 (22.2)	0.363
Chronic complications, n (%)						
Hypertension	97 (29.5)	87 (26.8)	0.440	63 (28.0)	64 (28.4)	0.917
ACEI/ARB use	35 (10.6)	32 (9.8)	0.738	18 (8.0)	17 (7.6%)	0.860
Diabetes mellitus	58 (17.6)	59 (18.2)	0.861	43 (19.1)	47 (20.9)	0.637
Cardiovascular disease	28 (8.5)	23 (7.1)	0.494	21 (9.3)	18 (8.0)	0.615
Chronic kidney disease	5 (1.5)	3 (0.9)	0.725	3 (1.3)	2 (0.9)	1.000
Cerebrovascular disease	22 (6.7)	13 (4.0)	0.127	12 (5.3)	10 (4.4)	0.662
Cirrhosis	87 (26.4)	75 (23.1)	0.319	50 (22.2)	54 (24.0)	0.655

Values are mean ± SD or number (percentage).

SD, standardized difference; UDCA, ursodeoxycholic acid; ACEI, angiotensin-converting enzyme inhibitor; ARB, angiotensin receptor blocker.

### Characteristics of the baseline laboratory variables and COVID-19 vaccination

In terms of liver function indicators, there were significant differences in γ-glutamyl transpeptidase and alkaline phosphatase between the two groups (p < 0.05), indicating that the UDCA group had poorer hepatic conditions. No difference was observed in blood routine counts, serum lipid parameters, glycosylated hemoglobin, and estimated glomerular filtration rate. The COVID-19 vaccination rate in the UDCA group was lower than in the control group (p < 0.001). In terms of vaccination doses, the control group received a higher proportion of the three doses (**Table 2**).

**Table 2:** Comparison of the baseline laboratory variables and vaccination.

Characteristics	Control (n = 225)	UDCA (n = 225)	p-Value
Liver function indicators			
ALT (U/L)	23.0 (15.5, 37.0)	21.0 (16.0, 35.0)	0.503
AST (U/L)	26.0 (20.0, 38.0)	28.0 (22.0, 37.0)	0.228
GGT (U/L)	30.0 (13.0, 58.0)	45.0 (20.0, 118.5)	<0.001
ALP (U/L)	79.0 (62.0, 107.0)	99.0 (78.5, 139.5)	<0.001
Total bilirubin (μmol/L)	15.0 (11.0, 23.5)	16.0 (12.0, 28.0)	0.193
Albumin (g/L)	44.0 (37.0, 47.0)	42.0 (38.0, 46.0)	0.129
Serum lipid parameters			
Total cholesterol (mmoI/L)	4.2 (3.4, 5.0)	4.2 (3.6, 5.3)	0.112
Triglyceride (mmoI/L)	1.1 (0.8, 1.6)	1.2 (0.9, 1.6)	0.375
Renal function parameter			
eGFR (ml/min/1.73 m^2^)	98.3 ± 13.8	96.4 ± 13.7	0.147
Glucose indicator			
HbA1c (%)	5.4 ± 0.8	5.5 ± 0.8	0.162
Blood routine index			
White blood cell (10^9^/L)	5.2 ± 1.9	5.2 ± 2.4	0.875
Hemoglobin (g/L)	138.4 ± 19.3	126.8 ± 23.3	0.134
Platelet (10^9^/L)	180.3 ± 89.9	172.4 ± 96.6	0.368
COVID-19 vaccination, n (%)			
Unvaccinated	79 (35.1)	125 (55.6)	<0.001
One dose	6 (2.7)	3 (1.3)	0.503
Two doses	19 (8.4)	22 (9.8)	0.744
Three doses	118 (52.4)	75 (33.3)	<0.001
Four doses	3 (1.3)	0	0.248

Values are median (interquartile range) or mean ± SD.

COVID-19, coronavirus disease 2019; UDCA, ursodeoxycholic acid; ALT, alanine aminotransferase; AST, aspartate aminotransferase; GGT, γ-glutamyl transpeptidase; ALP, alkaline phosphatase; eGFR, estimated glomerular filtration rate; HbA1c, glycosylated hemoglobin.

### Risk factors of COVID-19 infection in the matched cohorts

**Figure 1:**
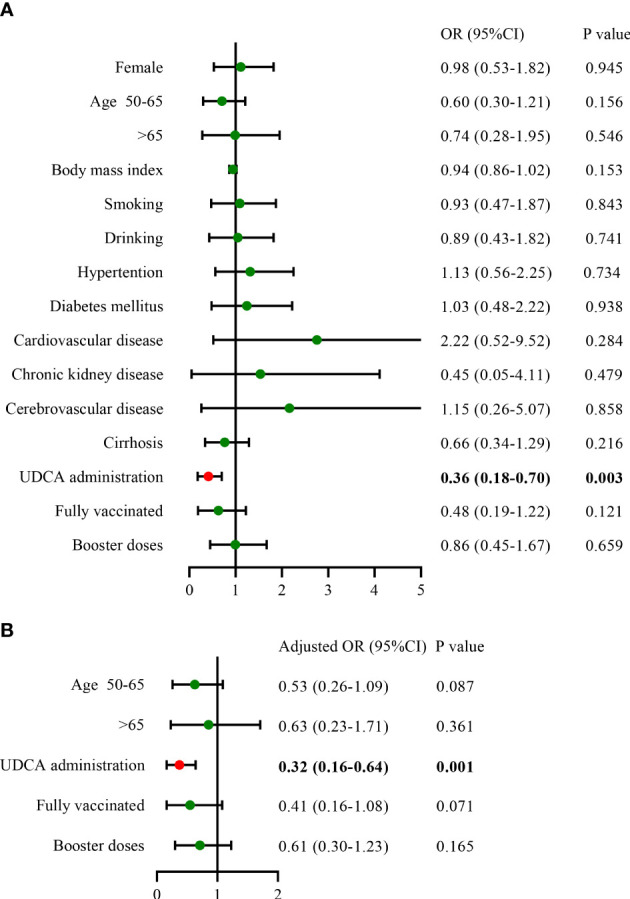
Influencing factors of COVID-19 infection in the matched cohorts **(A)**, univariate analysis; **(B)**, multivariate analysis) (n = 450). COVID-19, coronavirus disease 2019; OR, odds ratio; CI, confidence interval; UDCA, ursodeoxycholic acid.

**Figure 2:**
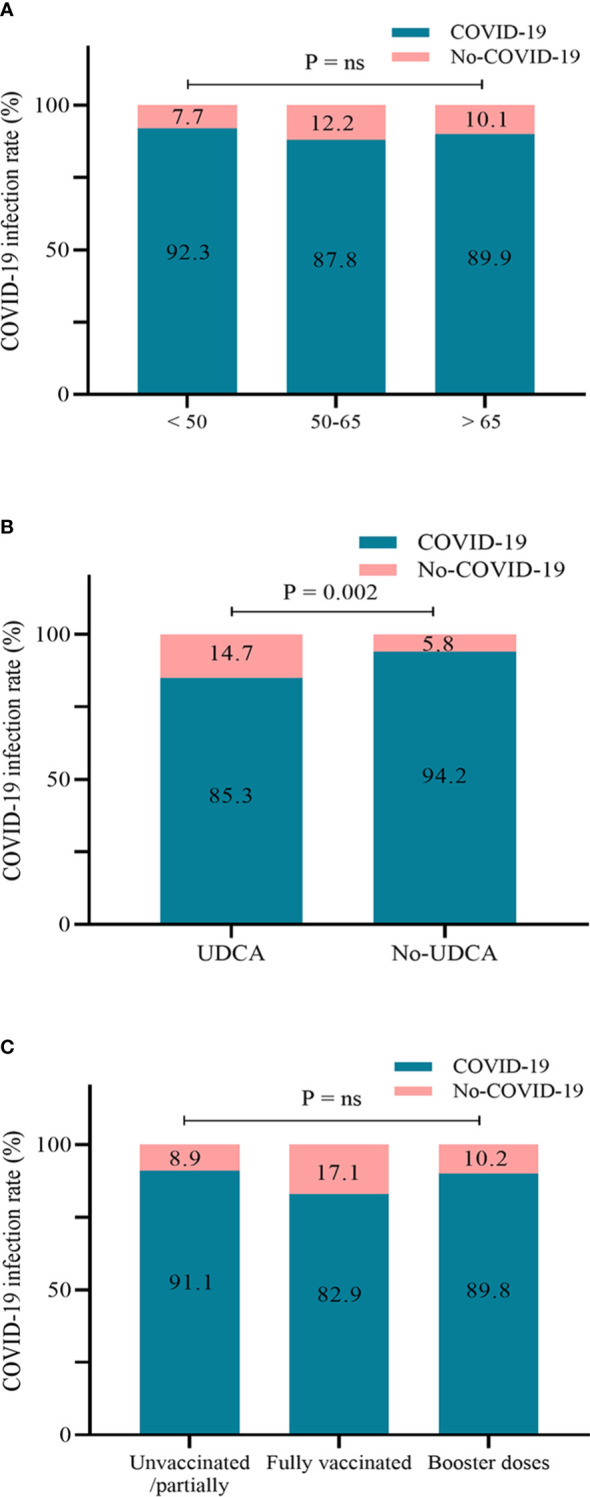
Infection rate of COVID-19 according to age groups **(A)**, UDCA use **(B)**, and vaccination status **(C)** (n = 450). COVID-19, coronavirus disease 2019; UDCA, ursodeoxycholic acid.

We included 450 matched patients in the logistic regression analysis. Through multivariate analysis, UDCA administration had an OR of 0.32 (95%CI: 0.16–0.64, p = 0.001), a protective factor against SARS-CoV-2 infection. A total of 404 patients developed COVID-19, with an overall infection rate of 89.8%. We further assessed the impact of UDCA use, age stratification, and vaccination status on the incidence of COVID-19. In the UDCA group, the rate of COVID-19 infection was remarkably lower than in the control group (85.3% *vs.* 94.2%, p = 0.002). However, no difference in the incidence was observed between age groups and vaccination groups (p > 0.05), as shown in **Figures 1**, **2**. Further analysis showed that patients with COVID-19 had similar baseline characteristics to these uninfected individuals, as shown in **Supplementary Table 1**.

### Clinical symptoms and manifestations of COVID-19 infection in the matched cohorts

Most of the patients chose the SARS-CoV-2 antigen test. The UDCA group outperformed the control group in terms of signs and symptoms of SARS-CoV-2 infection. The maximum body temperature in the control group was higher than that in the UDCA group (38.5 *vs.* 38.7, p < 0.001) (**Table 3**). The UDCA group presented lower proportions of fever, shivering, cough, pharyngalgia, taste and smell dysfunction, and dyspnea than the control group (p < 0.05). Moreover, most of the mild infections occurred in the UDCA group (80.0% *vs.* 72.0%, p = 0.047). Although there was no significant difference in the proportion of moderate and severe cases between the two groups, their frequency was higher in the control group. Notably, there were no critical cases in either group.

**Table 3:** Clinical symptoms and manifestations of SARS-CoV-2 infection between the matched groups.

Characteristics	Control (n = 225)	UDCA (n = 225)	OR (95%CI)	p-Value
Infection test				
SARS-CoV-2 antigen test	167 (74.2)	173 (76.9)	0.86 (1.28–1.33)	0.510
SARS-CoV-2 ribonucleic acid test	58 (25.8)	52 (23.1)	–	–
General symptoms				
Fever, n (%)	205 (91.1)	182 (80.9)	2.42 (1.37–4.27)	0.002
Tmax (°C), median (IQR)	38.7 (38.4, 39.0)	38.5 (38.0, 39.0)	–	<0.001
Shivering, n (%)	69 (30.7)	31 (13.8)	2.77 (1.72–4.44)	<0.001
Cough, n (%)	160 (71.1)	125 (55.6)	1.97 (1.33–2.91)	0.001
Nasal obstruction, n (%)	21 (9.3)	17 (7.6)	1.26 (0.66–2.46)	0.498
Headache, n (%)	27 (12.0)	30 (13.3)	0.89 (0.51–1.55)	0.671
Pharyngalgia, n (%)	127 (56.4)	86 (38.2)	2.09 (1.44–3.05)	<0.001
Muscular ache, n (%)	102 (45.3)	82 (36.4)	1.45 (0.99–2.11)	0.055
Taste and smell dysfunction, n (%)	93 (41.3)	53 (23.6)	2.29 (1.52–3.44)	<0.001
Fatigue, n (%)	108 (48.0)	101 (44.9)	1.13 (0.78–1.64)	0.508
Dyspnea, n (%)	11 (4.9)	3 (1.3)	3.80 (1.05–13.89)	0.030
Nausea, n (%)	8 (3.6)	9 (4.0)	0.88 (0.34–2.34)	0.805
Diarrhea, n (%)	3 (1.3)	3 (1.3)	1.00 (0.20–5.00)	1.000
Liver disease exacerbation, n (%)				
Liver pain	5 (2.2)	3 (1.3)	1.68 (0.40–7.14)	0.724
Ascites	4 (1.7)	2 (0.9)	2.02 (0.36–11.11)	0.685
Edema	2 (0.9)	2 (0.9)	1.00 (0.32–8.21)	1.000
Clinical diagnosis, n (%)				
Mild	162 (72.0)	182 (80.0)	0.64 (0.42–0.99)	0.047
Moderate	45 (20.0)	9 (4.0)	5.99 (2.86–12.66)	<0.001
Severe	5 (2.2)	1 (0.4)	5.10 (0.59–43.48)	0.216
Critical	0	0	–	–
Therapy, n (%)				
Home medication	183 (81.3)	150 (66.7)	2.18 (1.41–3.37)	<0.001
Outpatient	4 (1.7)	5 (2.2)	0.80 (0.21–3.00)	1.000
Hospital	5 (2.2)	3 (1.3)	1.68 (0.40–7.14)	0.724
No medication	20 (8.9)	34 (15.1)	0.55 (0.30–0.99)	0.042
Outcomes				
Recovery (days), median (IQR)	7 (5, 10)	5 (4, 7)	–	<0.001
Death, n (%)	0	0	–	–

Values are number (percentage) or median (interquartile range).

SARS-CoV-2, respiratory syndrome coronavirus-2; UDCA, ursodeoxycholic acid; OR, odds ratio; CI, confidence interval; IQR, interquartile range.

### Progression of the chronic liver disease and treatment during the infection

Regarding the effect of SARS-CoV-2 infection on underlying hepatic disease, four patients in the UDCA group and five in the control group suffered varying degrees of liver disease progression, such as liver pain, ascites, and edema. We also analyzed the management of the two groups during infection. The majority chose home medication, and the control group had more cases of self-medication than the UDCA group (66.7% *vs.* 81.3%, p < 0.001). There was no significant difference in the proportion of outpatient and inpatient visits between the two groups (p > 0.05). Specifically, nine patients were seen in the outpatient clinic mainly because of unremitting symptoms of COVID-19. In the control group, two patients were hospitalized due to advanced age and liver disease progression, and three inpatients had worsening infection symptoms. The principal reasons for hospitalization in the UDCA group were acute decompensation of cirrhosis (two cases) and severe COVID-19 infection (one case). In addition, UDCA had the advantage of not requiring medication (15.1% *vs.* 8.9%, p = 0.042) and a shorter time from infection to recovery (5 *vs.* 7 days, p < 0.001). Fortunately, there were no deaths in either group (**Table 3**).

### Factors associated with recovery time of COVID-19 infection in UDCA group

**Figure 3:**
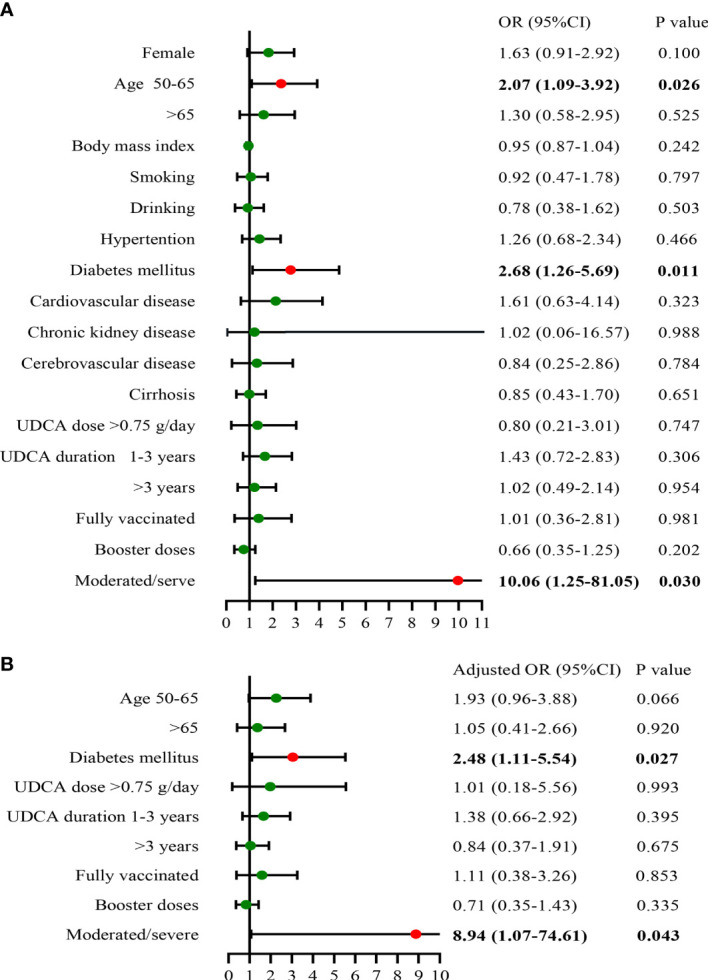
Risk factors of time from infection to recovery in UDCA users **(A)**, univariate analysis; **(B)**, multivariate analysis) (n = 190). UDCA, ursodeoxycholic acid; OR, odds ratio; CI, confidence interval.

**Figure 4:**
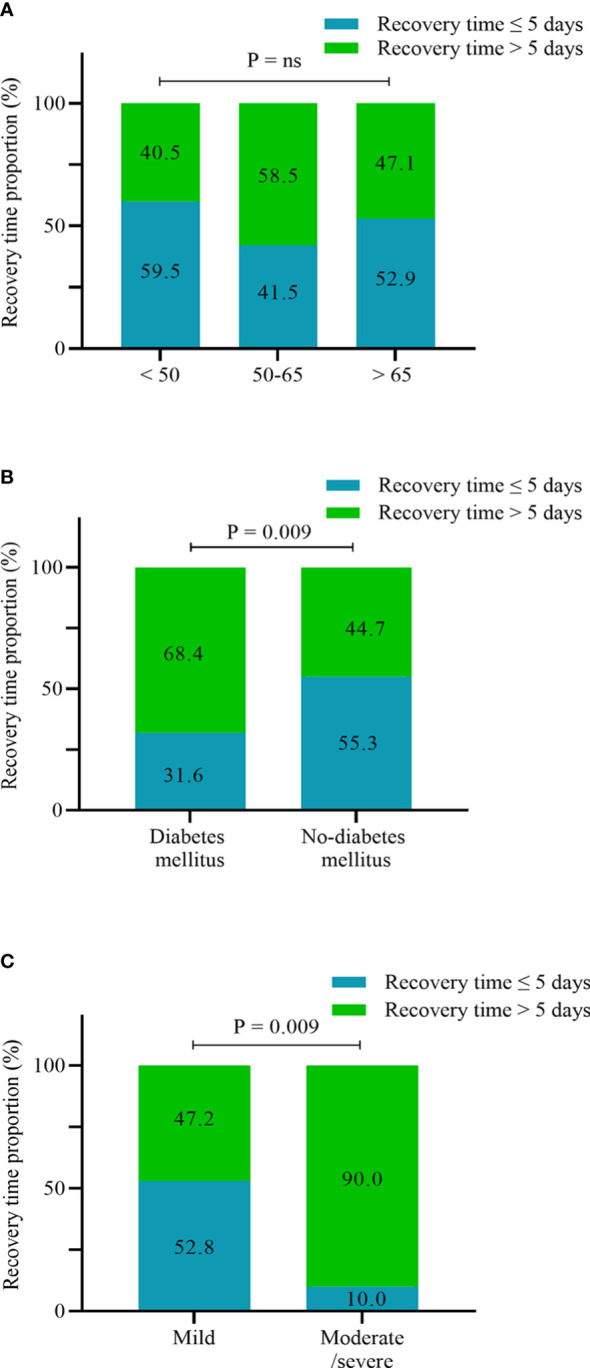
Proportion of recovery time ≤5 days and >5 days according to age stratification **(A)**, diabetes mellitus **(B)**, and infection severity **(C)** (n = 190).

We next analyzed the potential risk factors for the recovery time of SARS-CoV-2 infection in the UDCA group (n = 190), excluding two asymptomatic infection cases. The median time from infection to recovery was 5 days (interquartile range, 4–7 days) (**Table 3**), and we defined more than 5 days as a long recovery time. Univariate analysis showed that patients aged between 50 and 65 years, combined diabetes mellitus, and moderate/severe SARS-CoV-2 infection were associated with a longer course (**Figure 3**). By multivariable adjustment analysis, we identified that diabetes mellitus (OR: 2.48, 95%CI: 1.11–5.54, p = 0.027) and moderate/severe infection (OR: 8.94, 95%CI: 1.07–74.61, p = 0.043) increased the risk of prolonged time from infection to recovery. Furthermore, subgroup analysis showed that the recovery time was more likely to be longer than 5 days in the diabetes mellitus group and moderate/severe infection group (68.4% *vs.* 44.7%, p = 0.009; 90.0% *vs.* 47.2%, p = 0.009, respectively) (**Figure 4**). Although there was no significant difference in recovery time between age groups, the older patients tended to recover slowly.

### Influence of antihypertensive drugs treatment on COVID-19

Given that the angiotensin-converting enzyme inhibitors (ACEIs) and angiotensin-II receptor blockers (ARB) could augment ACE2 expression, we analyzed whether ACEI/ARB treatment would have an impact on the incidence and recovery time of COVID-19 in patients with chronic liver disease complicated by hypertension (n = 127). These patients were divided into two groups according to whether they were on ACEI/ARB or not. However, there was no significant difference in incidence and recovery time between the two groups (p > 0.05) (**Supplementary Figure 1**). We further analyzed the effect of ACEI/ARB combined with UDCA treatment on COVID-19 (n = 35) and found that the infection rate and recovery time were quantitatively lower in UDCA users than in those treated without UDCA (p > 0.05), as shown in **Supplementary Figure 2**.

## Discussion

In the present study, we found that 1) patients with chronic liver disease were generally susceptible to SARS-CoV-2 infection, with a total of 404 (89.8%) developing COVID-19; 2) UDCA was a protective factor against SARS-CoV-2 infection, with a significantly reduced incidence in UDCA users; 3) for patients with chronic liver disease, UDCA administration may have some benefits in improving clinical outcomes, such as reducing symptoms and severity and shortening the time from infection to recovery; 4) diabetes mellitus and moderate/severe infection were associated with longer recovery time in UDCA users rather than UDCA dose and duration.

Although the mortality from SARS-CoV-2 infection has declined sharply with the optimization of COVID-19 management, the highly transmissible SARS-CoV-2 variants continue to threaten global health (Raman et al., 2021). A recent study based on estimates from a big data model showed that approximately 900 million people (64%) in China were infected with SARS-CoV-2 as of 11 January 2023 (https://m.163.com/dy/article/HRVHIEFN0534I43Y.html). Preventing SARS-CoV-2 infection and mitigating harm are the common goals of all humankind (Wang et al., 2021). Antiviral treatment options, such as molnupiravir and paxlovid, improved clinical prognosis only in some of the specific cases, and cost and availability further hindered their clinical application (Lai et al., 2022; Wong et al., 2022). Vaccines also face rigorous challenges. The limited protective potency and the emergence of vaccine-resistant virus mutants render antibodies unable to provide broad-spectrum protection (Shrestha et al., 2022). Thus, there is an urgent need for novel prophylactic agents that reduce the risk of severe infection, are less susceptible to virus resistance, and are compatible with healthcare systems in low-income groups (Bartoszko et al., 2021). Recent experiment investigation has reported that the classic drug originally used for liver disease, UDCA, could protect against SARS-CoV-2 infection by downregulating ACE2 (Brevini et al., 2022). However, this finding needs further validation in clinical practice.

UDCA is mainly administered to patients with chronic liver disease, which could effectively treat hepatitis-related hepatic dysfunction and cholestatic liver disease (European Association for the Study of the Liver, 2017; Hu et al., 2019). We therefore investigated the impact of UDCA on COVID-19 in patients with hepatitis B and autoimmune liver disease in the era of epidemic prevention liberation. As hypothesized, we identified the association between UDCA administration and positive clinical prognosis following SARS-CoV-2 infection, including a reduction in infection rate and symptoms and a shortening of the time from infection to recovery. However, UDCA failed to completely inhibit SARS-CoV-2 infection; the incidence in the UDCA group was 85.4%. The reasons for the difference from the experimental findings were as follows (Brevini et al., 2022). First, the infection and severity of COVID-19 can be influenced by a number of uncontrollable confounding factors in clinical practice, such as complications and individual heterogeneity. Second, both the liberation of the epidemic and the high infectivity of the virus put individuals at risk of infection, including those with chronic liver disease (Suzuki et al., 2022). Moreover, considering that almost half of the patients in the UDCA group had not received COVID-19 vaccination, they may have less immunity to viral attacks (Chenchula et al., 2022). It was also possible that most of the individuals in the UDCA group received a lower dose of UDCA (75 mg/day) than the total dose in eight volunteers (15 mg·kg^−1^·day^−1^) reported by Brevini et al. (2022). Thus it did not have the full effect of blocking virus invasion. Notably, a small number of individuals with UDCA (14.7%) and without UDCA (5.8%) were free of infection. These “escaption” individuals were most likely patients with asymptomatic infections who did not receive regular SARS-CoV-2 ribonucleic acid or antigen testing. In addition, some people might minimize the chance of contact with the outside world and take great care to protect themselves, thereby significantly reducing the infection risk. We cannot rule out the possibility that there are truly uninfected populations that successfully resist SARS-CoV-2 infection because of their strong immunity. They may eventually suffer SARS-CoV-2 infection as the virus continues to spread.

Importantly, we also observed that diabetes mellitus and moderate/severe infection evidently prolonged the time from infection to recovery in UDCA users. However, the dose and duration of UDCA were independent of the recovery time. The possible reasons for this were that some of the patients interrupted the UDCA administration during the infection, or the relatively low daily dose failed to provide adequate protection against COVID-19. Several studies have suggested that diabetes mellitus, as a pro-inflammatory disease, increases the risk of severe SARS-CoV-2 infection and prolonged recovery (Onder et al., 2020; Al-Kuraishy et al., 2021). Furthermore, some of the patients were elderly and had multiple chronic comorbidities; they may be susceptible to moderate/severe infection and prolonged recovery time due to poor immunity and pro-inflammatory conditions (Li et al., 2022). Considering that the ACEI/ARB could increase ACE2 expression, we investigated the impact of these antihypertensive drugs on SARS-CoV-2 infection. However, ACEI/ARB treatment alone had no effect on the incidence and severity of COVID-19, which was consistent with previous studies (Hu et al., 2020; Li et al., 2021). Further analyzing the effect of ACEI/ARB combined with UDCA treatment on COVID-19, we found that the combination therapy slightly decreased the infection risk and recovery time. The failure of UDCA to counteract the ACEI/ARB-induced increase in ACE2 may be related to the small number of people studied. Most importantly, there were some discrepancies between the clinical practice and laboratory studies.

Since the COVID-19 pandemic, there has been growing evidence that patients with COVID-19 frequently present with hepatic damage (Chen et al., 2020; Moon et al., 2020). A retrospective study of 105 patients with chronic hepatitis B and SARS-CoV-2 revealed liver injury in 14 patients (13.3%) and acute-on-chronic liver failure in four patients (3.8%) (Zou et al., 2021). Data from the summary of early published studies reported that acute hepatic decompensation was a common presenting manifestation occurring in up to 46% of patients with cirrhosis, usually with new or deteriorating ascites and/or hepatic encephalopathy (Dufour et al., 2022). Contrary to previous studies, we found that patients were at relatively low risk of suffering liver disease progression following SARS-CoV-2 infection. Although further assessment of liver function indicators was lacking, the overall hepatic clinical manifestations in our patients were favorable. There were nine cases (2%) that experienced the exacerbation of hepatic conditions, such as liver pain and ascites, but none of the patients died. Undoubtedly, most of the available knowledge referred to prior studies conducted in the era preceding COVID-19 vaccination or the emergence of less virulent variants, including Delta and Omicron. On the one hand, vaccination greatly increased immunity and resistance to the virus. On the other hand, the attenuated viral pathogenicity also reduced organ damage and disease severity. Therefore, our results were not completely contradictory to those of previous studies, which were conducted in a different context.

Some limitations should be taken into account. First, the relatively small number of patients, and the fact that the study was conducted in a single center, means that the efficacy and safety of UDCA against COVID-19 need to be further validated in large-scale multi-center studies. Second, there was case selection bias due to the inclusion of different chronic liver diseases, including chronic hepatitis B and autoimmune liver disease. To address that, we controlled the confounding by performing a propensity score adjustment analysis using the nearest neighbor matching algorithm. However, there may be potential differences in the susceptibility to SARS-CoV-2 among various chronic liver diseases. The residual and unmeasured confounding probably persist, and our findings did not prove causation. Moreover, because most of the infected individuals did not seek medical attention, this study lacked the evaluation of laboratory indicators and imaging examination following infection. This may be related to the following reasons: 1) the liberation of the epidemic and the sudden increase in infections led to the shortage of medical resources; 2) most of the infections were mild and recovered spontaneously due to the diminished pathogenicity of SARS-CoV-2. Accordingly, we were unable to analyze the possible protective mechanisms of UDCA on COVID-19 due to the lack of patients’ blood samples. We were also unable to assess the effect of UDCA on viral shedding time, as few individuals undergo SARS-CoV-2 ribonucleic acid or antigen re-test during universal infection. Finally, it should be emphasized that the present study was based on self-reports rather than classically established COVID-19 by experimental investigation, which may affect the accuracy of the conclusions.

## Conclusion

Patients with chronic liver disease were generally susceptible to SARS-CoV-2 infection. UDCA had a protective effect on COVID-19, including reducing the infection risk, mitigating symptoms, and shortening the time from infection to recovery. The daily dose and duration of UDCA were independent of recovery time, whereas diabetes mellitus and moderate/severe infection may prolong the course of COVID-19 in UDCA users. Therefore, UDCA might be beneficial in preventing SARS-CoV-2 infection in patients with chronic liver disease, especially cholestasis and hepatic dysfunction. However, these findings need further corroboration and validation in other cohort samples and experimental investigations.

## Data availability statement

The original contributions presented in the study are included in the article/**Supplementary Material**. Further inquiries can be directed to the corresponding author.

## Ethics statement

The studies involving human participants were reviewed and approved by The Ethical Review Committee of the Beijing Ditan Hospital. The patients/participants provided their written informed consent to participate in this study. Written informed consent was obtained from the individual(s) for the publication of any potentially identifiable images or data included in this article.

## Author contributions

YLi was the first author of this study. Study design: YLi. Data collection: NZ. Analysis of data: YLin and XC. Drafting of the manuscript: YLi. Critical reversion of the manuscript: XL. Material and technical support, and study supervision: XL. All authors have read and approved the manuscript.

## Supplementary material

Supplementary Figure 1 Influence of ACEI/ARB treatment on COVID-19 infection **(A)**, and recovery time **(B)** in patients with chronic liver disease complicated by hypertension (n = 127). ACEI, angiotensin converting enzyme inhibitors; ARB, angiotensin-II receptor blockers; COVID-19, coronavirus disease 2019.

Supplementary Figure 2 Influence of the UDCA in combination with ACEI/ARB treatment on COVID-19 infection **(A)**, and recovery time **(B)** in patients with chronic liver disease complicated by hypertension (n = 35). UDCA, ursodeoxycholic acid; ACEI, angiotensin converting enzyme inhibitors; ARB, angiotensin-II receptor blockers; COVID-19, coronavirus disease 2019.
