# Supplementary figures and images for: Protective effect of ursodeoxycholic acid on COVID-19 in patients with chronic liver disease
Source: Front Cell Infect Microbiol. 2023 May 3;13:1178590. doi: 10.3389/fcimb.2023.1178590 (PMC10189063; doi:10.3389/fcimb.2023.1178590)

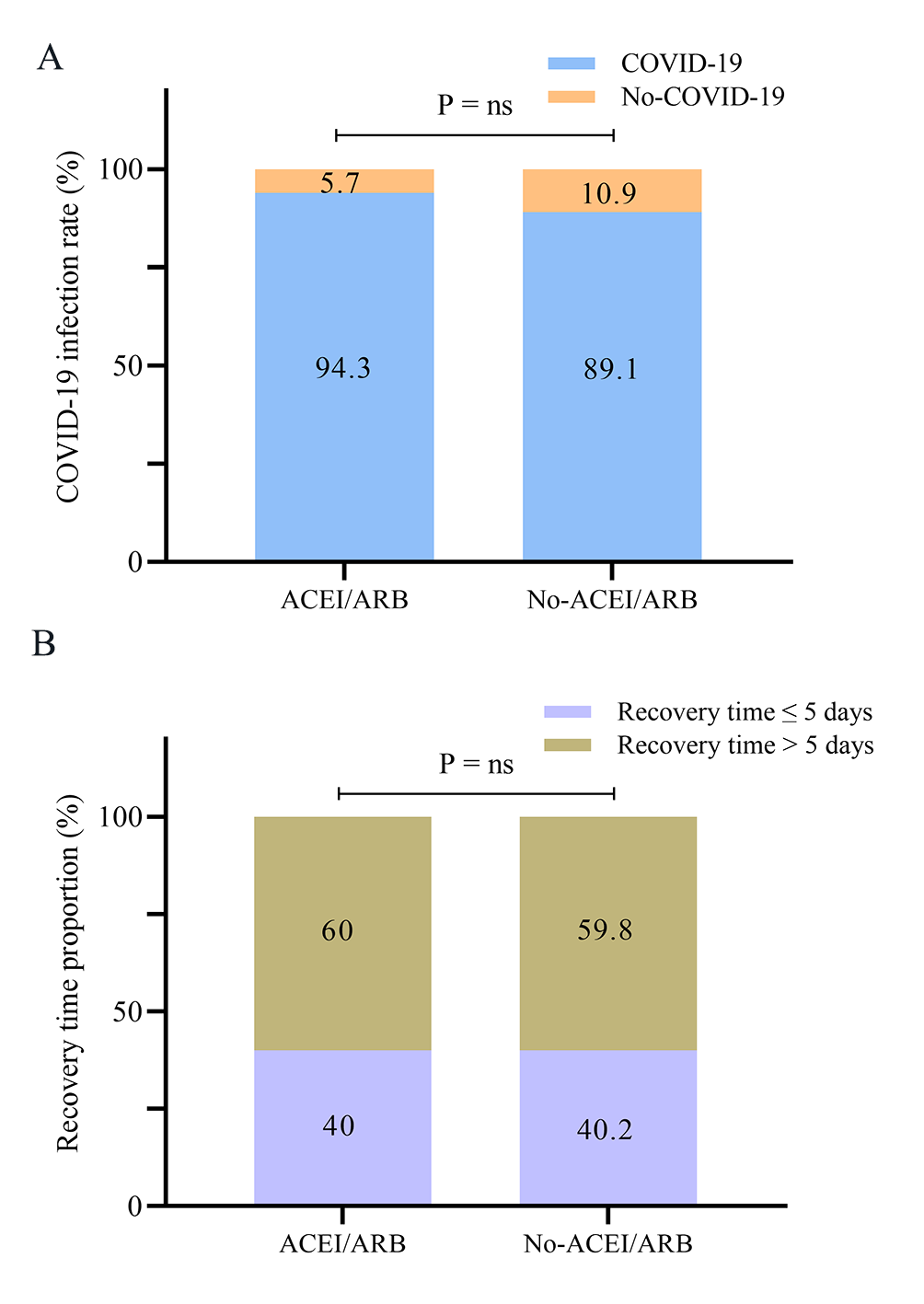

Supplement: Supplementary Figure 1 — Influence of ACEI/ARB treatment on COVID-19 infection (A), and recovery time (B) in patients with chronic liver disease complicated by hypertension (n = 127). ACEI, angiotensin converting enzyme inhibitors; ARB, angiotensin-II receptor blockers; COVID-19, coronavirus disease 2019. [file Image_1.tif]

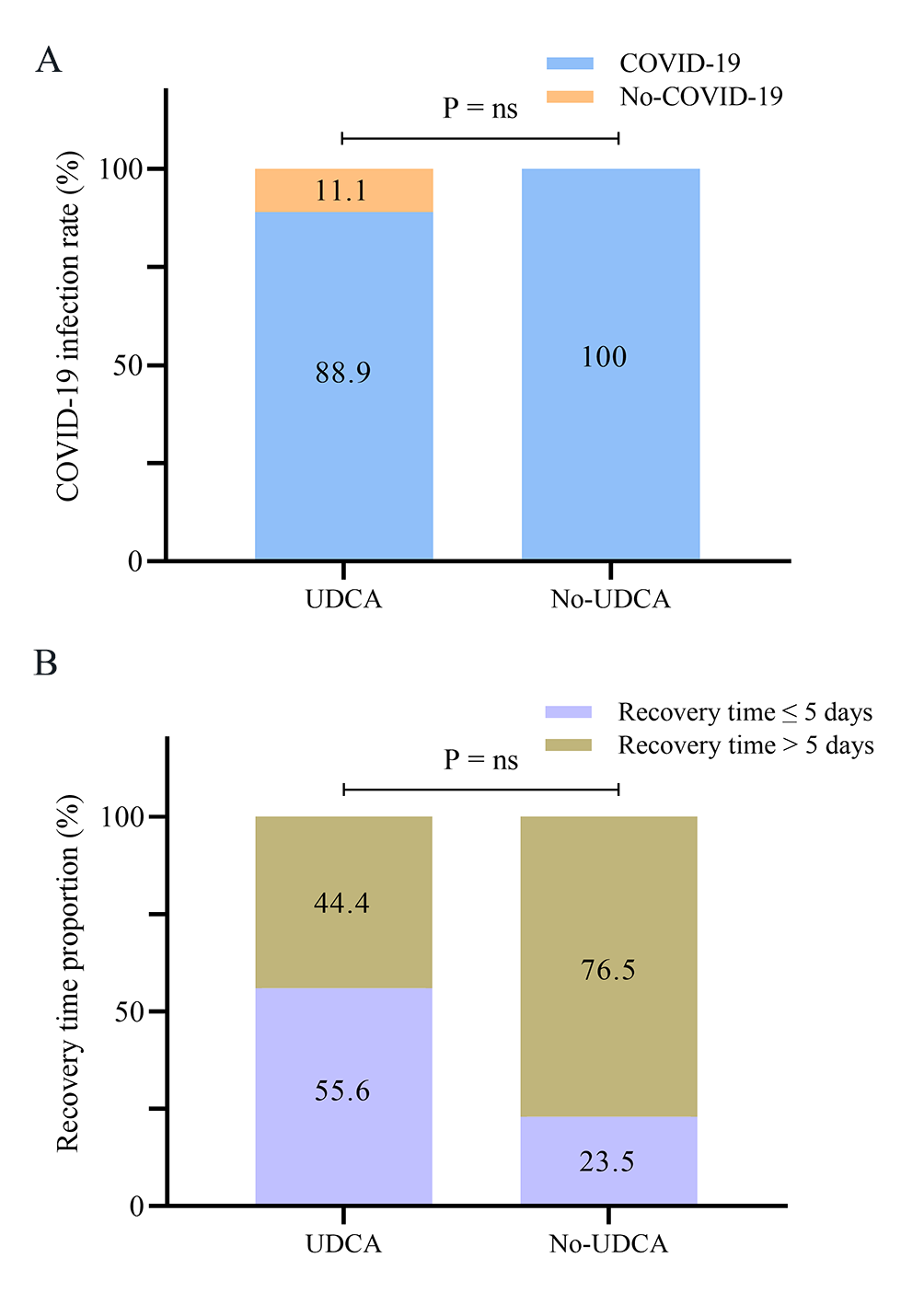

Supplement: Supplementary Figure 2 — Influence of the UDCA in combination with ACEI/ARB treatment on COVID-19 infection (A), and recovery time (B) in patients with chronic liver disease complicated by hypertension (n = 35). UDCA, ursodeoxycholic acid; ACEI, angiotensin converting enzyme inhibitors; ARB, angiotensin-II receptor blockers; COVID-19, coronavirus disease 2019. [file Image_2.tif]
