# Supplementary material for: Protective effect of ursodeoxycholic acid on COVID-19 in patients with chronic liver disease
Source: Front Cell Infect Microbiol. 2023 May 3;13:1178590. doi: 10.3389/fcimb.2023.1178590 (PMC10189063; doi:10.3389/fcimb.2023.1178590)
Supplement: Supplementary file 3 [file Table_1.docx]

| Characteristics | Infected  (n = 404) | Uninfected  (n = 46) | P value |
| --- | --- | --- | --- |
| Personal history |  |  |  |
| Age (years) | 53.2 ± 11.5 | 54.6 ± 10.6 | 0.433 |
| Body mass index (kg/m^2^) | 23.8 ± 3.2 | 24.5 ± 3.6 | 0.152 |
| Female, n (%) | 235 (58.2) | 27 (58.7) | 0.945 |
| Smoking, n (%) | 100 (24.8) | 12 (26.1) | 0.843 |
| Drinking, n (%) | 88 (21.8) | 11 (23.9) | 0.741 |
| Chronic complications, n (%) | 27 (12.0) | 30 (13.3) | 0.671 |
| Hypertension | 115 (28.5) | 12 (26.1) | 0.734 |
| Diabetes mellitus | 81 (20.0) | 9 (19.6) | 0.938 |
| Cardiovascular disease | 37 (9.2) | 2 (4.3) | 0.407 |
| Chronic kidney disease | 4 (1.0) | 1 (2.2) | 0.418 |
| Cerebrovascular disease | 20 (5.0) | 2 (4.3) | 1.000 |
| Cirrhosis | 90 (22.3) | 14 (30.4) | 0.214 |

**Supplementary Table 1 Comparison of baseline characteristics between infected and uninfected individuals**
